# An Improved Ratiometric FRET Biosensor with Higher Affinity for Extracellular ATP

**DOI:** 10.3390/s25185903

**Published:** 2025-09-21

**Authors:** Autumn Cholger, Jason M. Conley, Elaine Colomb, Olivia de Cuba, Jacob Kress, Mathew Tantama

**Affiliations:** 1 Department of Chemistry & Interdisciplinary Life Science Program, Purdue University, West Lafayette, IN 47907, USA; 2 Department of Chemistry & Biochemistry Program, Wellesley College, Wellesley, MA 02481, USA

**Keywords:** extracellular ATP, purinergic, FRET biosensor, fluorescent protein

## Abstract

Adenosine triphosphate (ATP) is readily released into the extracellular space as an autocrine and paracrine purinergic signaling molecule. We originally reported a genetically encoded, fluorescent protein-based Förster Resonance Energy Transfer (FRET) biosensor that can detect micromolar levels of extracellular ATP. Through mutagenesis of the ATP binding site and optimization of cell-surface display, here we report the development of a second-generation biosensor called ECATS2 with greater than three-fold higher affinity for extracellular ATP. We found that the tether length between the FRET biosensor and the cell surface anchor is critical to optimization of its performance. Furthermore, we demonstrate that the improved sensor can detect extracellular ATP release upon hypoosmotic stress in cultured astrocytes. This new sensor contributes an improved tool for the ratiometric detection of extracellular ATP dynamics and purinergic signaling.

## 1. Introduction

Extracellular ATP is an important purinergic signal that can activate ionotropic and metabotropic receptors during normal intercellular signaling as well as during injury and immune response [1,2]. For example, purinergic signaling plays a role in the development and resolution of edema-associated inflammation or when osmotic stress is present from tissue swelling [3,4,5,6,7]. It has been demonstrated that extracellular ATP is released upon hypoosmotic stress in both cell lines and primary cells such as astrocytes [4,5,6,7], and furthermore extracellular ATP is a key signal regulating microglial response to injuries in the brain [8,9].

However, a major challenge in the study of purinergic signaling is that the concentrations of extracellular ATP are extremely low compared to the millimolar concentrations of ATP found in the cytosol. For example, some P2X ion channels and P2Y G-protein coupled receptors are activated by as little as 0.1–1 µM ATP, indicating that physiologically relevant ATP levels may only fluctuate from nanomolar to micromolar levels [2,10]. As a consequence, biosensors to detect extracellular dynamics must exhibit high affinity for ATP.

To this end, several biochemical and biophysical methods have been used over the decades to detect purinergic signaling [11,12,13]. For example, microelectrodes have been used to successfully measure tissue levels of ATP, though ATP released from tissue damage during electrode insertion can be a confounding factor [14,15]. Fluorophore-labeled ATP analogues have been used to visualize vesicular release, but this does require loading of secretory vesicles [16]. Engineered purinergic sniffer cells have been used extensively to measure ATP release from astrocytes, though the technique can be challenging [17,18,19,20]. Luciferase imaging [21,22], and, in particular, imaging of membrane-tethered luciferase [23,24,25,26], has greatly facilitated the understanding of real-time purinergic dynamics, though light production is often low [27]. Alternatively, the use of bright fluorescent biosensors has shown increasing promise for the detection of low levels of extracellular ATP [11].

**Figure 1:**
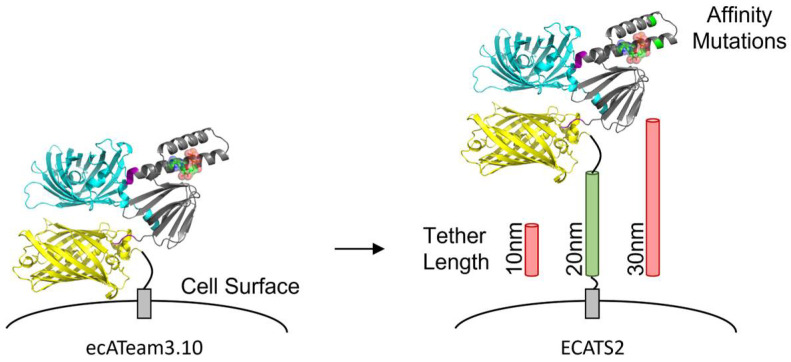
Engineering of second-generation extracellular ATP sensor ECATS2. The original ecATeam3.10 sensor is attached to the cell surface anchor by only a short peptide. The second-generation ECATS2 has affinity mutations at the ATP binding site and an optimized tether length.

We originally developed a genetically encoded, ratiometric biosensor of extracellular ATP using a two-color fluorescent protein FRET pair (Figure 1 and Figure S1) [28], and subsequently others developed non-ratiometric single-color biosensors using a circularly permuted green fluorescent protein (cpGFP) [29,30]. The single-color cpGFP-based biosensors have high dynamic range and are superb for detecting ATP release events. Although FRET biosensors generally have more limited dynamic range, their ratiometric readout offers potential advantages for comparison of longitudinal or parallel experiments because they are intrinsically normalized for biosensor expression level [28,31]. However, all of these current sensors optimally respond to 10–100 µM ATP release. Therefore, we sought to increase the affinity of our FRET biosensor through mutagenesis of the ATP binding site and optimization of its cell surface attachment.

## 2. Materials and Methods

***Molecular Biology.*** Mutations were introduced into ecATeam3.10 using the NEB Q5 Site-Directed Mutagenesis kit, and all other cloning was carried out using NEB Gibson/HiFi Assembly or restriction enzyme cloning (NEB, Ipswich, MA, USA). E/RK linkers were a kind gift from S. Sivaramakrishnan, and Pink Flamindo was a gift from Tetsuya Kitaguchi (Addgene plasmid #102356; http://n2t.net/addgene:102356; RRID:Addgene_102356). Adenovirus expression vectors were generated using the pAd/CMV/V5-DEST vector and Gateway cloning according to the manufacturer’s instructions (Invitrogen, Carlsbad, CA, USA).

***Cell Culture.*** Neuro2A and HEK293A cell lines were maintained in DMEM supplemented with 10% cosmic calf serum. Cells were transfected using Effectene (Qiagen, Boston, MA, USA) or by the calcium phosphate method. For biosensor comparisons, ecATeam3.10 plasmid was co-transfected with excess H2B-mApple plasmid mApple-H2B-6 was a gift from Michael Davidson (Addgene plasmid # 54908; http://n2t.net/addgene:54908; RRID:Addgene_54908) to ensure all ecATeam3.10-expressing cells were marked for identification with H2B-mApple (Figure S2). For Adenovirus production, the Life Technologies ViraPower Adenovirus Expression System (Invitrogen, Carlsbad, CA, USA) was used according to the manufacturer’s instructions. Cortical astrocytes were isolated from P1 C57BL/6 pups, and all animal procedures were approved by and performed in accordance with guidelines by the Purdue Animal Care and Use Committee. Cortical astrocytes were cultured in DMEM/F12 supplemented with 10% cosmic calf serum and penicillin-streptomycin and transduced on day 3 in vitro (Gibco, Thermo Fisher Scientific, Waltham, MA, USA).

***Microscopy***. Live-cell microscopy was carried out using an Olympus IX83 inverted fluorescence microscope controlled using Andor iQ3 software with a 20X/0.75 NA objective excited by a Lumencor SpectraX light engine and equipped with an Andor Zyla 4.2 sCMOS camera. A multiband dichroic (ET-ECFP/EYFP/mCherry, Chroma 69008bs) was used in combination with different bandpass exciters and emitters for each channel: cyan—438/29 nm and 470/24 nm; yellow—510/10 nm and 540/30 nm; sensitized FRET emission—438/29 nm and 540/30 nm; red—575/25 nm and 631/28 nm (Evident Scientific, Waltham, MA, USA).

Isotonic imaging solution was composed of 15 mM HEPES, 1.25 mM NaH_2_PO_4_, 10 mM glucose, 130 mM NaCl, 3 mM KCl, 2 mM CaCl_2_, 1 mM MgCl_2_, and 3 mM NaHCO_3_ (pH 7.3), and hypoosmotic stress was induced by lowering the NaCl concentration to 65 mM. For primary astrocytes, imaging solutions were additionally supplemented with 0.2 mM sodium pyruvate and 0.5 mM GlutaMAX. All cells were equilibrated at room temperature for at least 20 min in isotonic solution prior to imaging. Imaging was carried out at room temperature with continuous perfusion at a flow rate of 1.5 mL/min for at least 3 to 5 independent cultures.

Images were analyzed using ImageJ/FIJI. Mean background intensities were measured for each channel in each field of view for background subtraction, and cell masks were created using a minimum threshold of at least two times the mean background intensity. As described previously [28], regions of interest were carefully drawn around each cell membrane to exclude intracellular background. Ratio images were calculated by pixel-by-pixel division, and regions of interest that isolated surface (ecATeam) or whole cells (Pink Flamindo) were manually drawn. Statistical analysis and fitting were carried out using OriginPro 2017 software, and two-tailed students’ *t*-test comparisons were deemed significant for *p* < 0.05 when comparing means.

***Software.*** The protein structure shown in Figure 1 was generated using the AlphaFold server (alphafoldserver.com, accessed on 17 July 2025). The protein sequence for ATeam3.10 was used as the input, and MgATP was added as a ligand. PyMol 3.1.3 was used to generate an image of the AlphaFold model structure, and the final cartoon figure was created using Microsoft PowerPoint 365.

## 3. Results

### 3.1. Mutation of the ATP Binding Site

Previously, we engineered ecATeam3.10, which uses the ATP synthase ε subunit from *Bacillus* sp. PS3 flanked by the cyan and yellow fluorescent proteins mseCFP and mVenus [28,32]. While the wildtype ε subunit exhibits micromolar affinity for ATP [28,32,33], Kato-Yamada reported that the combined mutations R103A and R115A near the ATP binding site increase the affinity to an apparent dissociation constant of approximately 50 nM (Figure S1) [34]. Because the presence of the attached fluorescent proteins is known to modulate the apparent affinity of the ε subunit [32,33,35], we tested the effect of the R103A/R115A double mutation in the context of the ATeam3.10 biosensor. Using purified proteins in solution, we found that compared to the original ATeam3.10 [32] the double mutant exhibits a 4-fold increase in ATP affinity at room temperature with an apparent dissociation constant of 0.2 µM, slightly higher than the ε subunit alone [34]. We therefore installed the R103A/R115A double mutation in our extracellular ATP biosensor and compared the ATP dose responses between wildtype ecATeam3.10 and double-mutant ecATeam3.10(A/A) when expressed on the surface of live cultured cells. For the initial screenings, we carried out an abbreviated 3-point ATP dose response assay to identify any obvious changes in affinity or dynamic range (defined as the change in sensitized emission ratio at saturation versus in the absence of ATP). The lowest concentration of 3 µM was chosen because this represents a low concentration of ATP near the original ecATeam3.10’s limit of detection, and the highest concentration of 300 µM ATP will saturate the sensor. To facilitate direct comparison of the biosensors, they were imaged simultaneously using separately transfected cells that were then mixed when grown on the imaging dish (Figure S2). The cells expressing the original ecATeam3.10 biosensor were co-transfected with H2B-mApple as a marker for identification.

**Figure 2:**
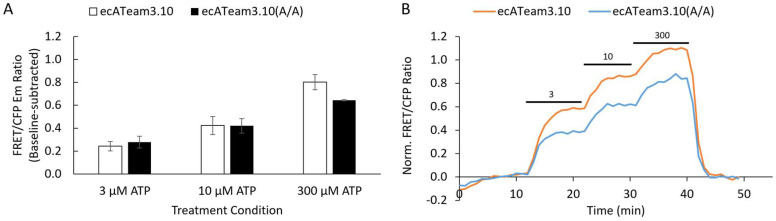
The R103A/R115A double-mutation alone does not increase the affinity for extracellular ATP. (**A**) Average response of cells to increasing concentrations of extracellular ATP for the original ecATeam3.10 (white, n = 31 cells) versus the ecATeam3.10(R103A/R115A) double mutant (black, n = 69 cells). Errors are standard errors of the mean (sem). (**B**) Representative single-cell responses in for the live-cell perfusion experiment. The original ecATeam3.10 in orange, and the ecATeam3.10(A/A) double mutant in blue. Black bars indicate the concentration of ATP being perfused in micromolar.

Surprisingly, we did not observe a change in affinity for the ecATeam3.10(A/A) double mutant compared the original ecATeam3.10, which exhibits an apparent dissociation constant of 12 µM (Figure 2). Instead, we observed that the double-mutant ecATeam3.10(A/A) actually suffers from a decreased dynamic range relative to the wildtype. We confirmed that ATP binding to the purified soluble sensing domain of the double mutant does not require magnesium, similar to what was reported for the wildtype *Bacillus* sp. PS3 ε subunit (Figure S3). We therefore suspected that a physical mechanism might mask the change in affinity. In particular, during the process of engineering the original ecATeam3.10, we observed a similar phenomenon in which tethering the ATeam FRET biosensor to the cell surface greatly diminished affinity and dynamic range compared to the soluble biosensor [28]. We therefore hypothesized that optimization of the cell surface tether might improve the double-mutant’s performance.

### 3.2. Optimization of Tether Length

The original ecATeam3.10 biosensor is displayed on the extracellular cell surface by the platelet-derived growth factor receptor (PDGFR) transmembrane domain, and it includes only a short 13-amino acid tether to the mVenus acceptor [28]. With this short tether, we suspected that the close proximity to the cell surface could cause steric restriction that limits the conformational dynamics of the FRET biosensing module. To test if increasing the distance from the membrane would improve the double-mutant biosensor, we replaced the original short tether with a 10 nm, 20 nm, or 30 nm length α-helical ER/K linker developed by Sivaramakrishnan and Spudich [36]. We chose the ER/K linkers because they were shown to be semi-rigid and we hypothesized the rigidity could help better hold the sensing module at a distance from the surface. In the future it may be interesting to explore different types of tethers, but in this study we worried that flexible linkers, such as a glycine-serine polypeptide or the EV linkers [37], might have too much flexibility and allow the sensing module to sit near the membrane, or they might themselves suffer from proteolytic cleavage.

**Figure 3:**
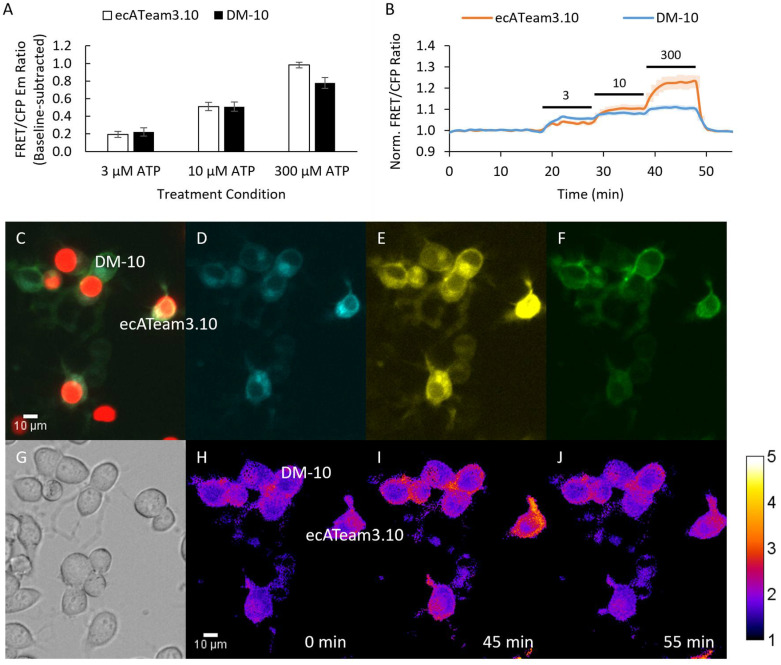
A 10 nm length tether does not improve biosensing by the double mutant. (**A**) Average response of cells to increasing concentrations of extracellular ATP for the original ecATeam3.10 (white, n = 6 cells) versus the DM-10 (black, n = 18 cells). (**B**) Population average for the original ecATeam3.10 (orange) and DM-10 (blue) time course responses. Bars indicate wash in of ATP at the µM concentration indicated. (**C**–**J**) Example images from the mixed-cell population imaging strategy that enables direct comparison of DM-10 and ecATeam3.10 performance. (**C**) Overlay of red fluorescence channel with (**D**) cyan, (**E**) yellow, and (**F**) FRET channels. Cells expressing ecATeam3.10 are clearly marked by H2B-mApple. (**G**) DIC image. (**H**–**J**) Ratio images pseudo-colored to the sensitized FRET emission–to-donor emission ratio at the indicated time points.

Each respective tether length in the double mutant background (“DM-10”, “DM-20”, or “DM-30”) was directly compared to the original ecATeam3.10. To do this, we mixed two separately transfected sets of cells together as we had done for the initial double mutant. One set of cells was co-transfected with the original ecATeam3.10 and nuclear-localized H2B-mApple as a marker. A second set of cells was transfected with one of the double-mutant tether length variants without H2B-mApple, and then the two sets of cells were mixed and plated together for imaging experiments (Figure 3 and Figure S2). The strength of this approach is that we can image two biosensors simultaneously to directly compare responses under the same experiment conditions. However, in this approach we are limited to pairwise comparisons. We therefore compared each respective linker variant to the original ecATeam3.10 because, ultimately, our primary goal was to engineer a second-generation biosensor that showed improvement over the original. During imaging, cells were perfused with increasing extracellular ATP concentrations followed by washout to demonstrate reversibility. Notably, either treatment with apyrase or simple washout after ATP addition was able to reverse sensor responses to baseline levels, including for the final chosen sensor in cell lines and primary cells, and so washout was used for the experiments reported here.

Interestingly, the 10 nm (DM-10) tether length candidate did not show improvement compared to the original ecATeam3.10 (Figure 3). Rather, DM-10 still suffers a decreased dynamic range similar to the ecATeam3.10(A/A) double mutant, suggesting that the 10 nm tether length is not long enough to relieve steric hindrance by the cell membrane.

**Figure 4:**
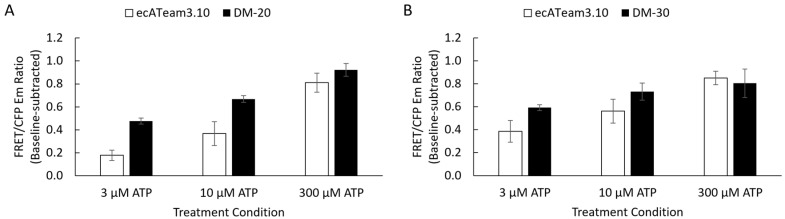
Initial screening showed the 20 nm and 30 nm tether lengths improved sensitivity to a lower extracellular ATP concentration. Simultaneous imaging enabled direct pairwise comparisons of each respective tether length against the original ecATeam3.10. Average responses to extracellular ATP concentrations are summarized as mean ± sem for (**A**) DM-20 (black, n = 31 cells) versus ecATeam3.10 (white, n = 22 cells) and (**B**) DM-30 (black, n = 56 cells) versus ecATeam3.10 (white, n = 29 cells).

**Figure 5:**
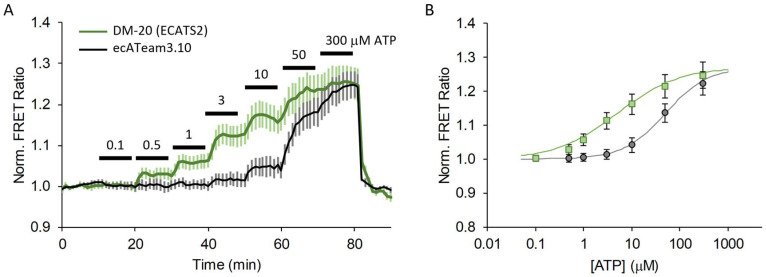
A 20 nm length tether greatly increases extracellular ATP affinity. (**A**) Average response to increasing extracellular ATP concentration on cells expressing the original ecATeam3.10 (black, n = 12 cells) versus DM-20 (renamed ECATS2) (green, n = 18 cells), and (**B**) fitted dose–response curves.

In contrast, the 20 nm tether candidate (DM-20) and the 30 nm tether candidate (DM-30) exhibit improved sensitivity to ATP in the initial screenings, suggesting higher ATP affinity compared to ecATeam3.10 (Figure 4, Figure S4 and S5). DM-20 and DM-30 performed similarly, both showing greater responses to the lowest 3 µM extracellular ATP concentration compared to the respective experiment-paired ecATeam3.10 responses. DM-20 showed a slightly better response (2.7 ± 0.7 fold compared to paired ecATeam3.10) than DM-30 (1.5 ± 0.4 fold compared to paired ecATeam3.10) at this low ATP concentration. We therefore chose to pursue DM-20 because it provided the minimum tether length necessary for an improvement over the original ecATeam3.10. We subsequently carried out a full extracellular ATP dose–response curve on cells for DM-20 (Figure 5).

The DM-20 candidate exhibits a 3.2-fold increase in affinity—from 12 ± 5 µM for the original ecATeam3.10 down to 3.7 ± 0.6 µM for DM-20. Notably, it is very clear that DM-20 reproducibly responds to 0.5 µM ATP whereas ecATeam3.10 requires between 3–10 µM ATP for a response. From the fitted binding curves, DM-20 has a similar dynamic range of ~1.3-fold maximum increase in the FRET ratio upon ATP saturation when expressed on live cells. With its increased ATP affinity, we also expected that DM-20 would exhibit an increased ADP affinity. Indeed, compared to ecATeam3.10, DM-20 has a 2-fold lower apparent ADP dissociation constant of approximately 100 µM (Figure S6). However, this still represents a greater than 25-fold selectivity for ATP over ADP. Physiologically, extracellular ADP levels may rise to the tens of micromolar levels. For example, the P2Y_1_ receptor has an EC_50_ of 8 µM for ADP [2,10]. Thus, there may be some sensing crosstalk for extracellular ADP and ATP, but this would be expected to be minor given the substantial difference in sensitivity to the two nucleotides. We therefore chose DM-20 as our next-generation biosensor and named it “ECATS2” (for “ExtraCellular ATP Sensor generation 2”) and refer to it as such from here on.

### 3.3. Hypoosmotic ATP Release

**Figure 6:**
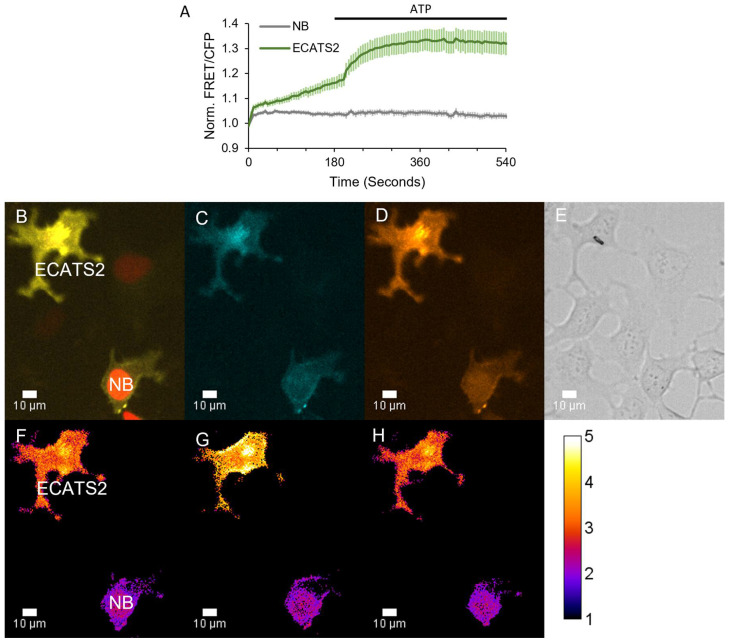
ECATS2 detects hypoosmotic stress-induced ATP release from HEK293 cells. (**A**) Average response from cells expressing ECATS2 versus the non-binding control mutant (“NB”). After equilibration in isotonic solution, hypoosmotic solution was washed in at the start of the experiment, eliciting a clear response from ECATS2 absent from the NB control. To verify maximum response, ATP was washed in at 180 s (black bar). Example images for the (**B**) overlay of the cyan, FRET, and red fluorescence channels. NB-expressing cells are marked for identification by H2B-mApple. (**C**) Cyan and (**D**) FRET individual channels. (**E**) DIC image. (**F**–**H**) Ratio images pseudo-colored to the sensitized FRET emission ratio (**F**) before hypoosmotic stress, (**G**) at peak response, and (**H**) at washout.

Next, we demonstrated that ECATS2 can detect the stimulated release of extracellular ATP from live cells. Purinergic signaling contributes to cellular volume regulation, and hypoosmotic stress can elicit ATP release to mediate homeostatic mechanisms [5,22,38,39]. As an initial test, ECATS2 was expressed on HEK293 cells, which we previously demonstrated release nucleotides upon hypoosmotic stress [40]. We also used a non-binding control sensor that harbors the R122K/R126K mutation that eliminates ATP affinity [32]. As described previously, cells were mixed and imaged together to enable the direct comparison of the ECATS2 versus non-binding control biosensor responses. Here, the non-binding control was co-expressed with H2B-mApple for identification. Hypoosmotic stress was induced by decreasing the osmolality of the imaging solution from approximately 300 to 150 mOsm/L. The ECATS2 biosensor reported a prompt increase in its sensitized FRET emission ratio upon hypoosmotic stress that was slowly reversed upon washout with isotonic imaging solution (Figure 6). We did observe a reproducible imaging artifact at the point of solution switching for both ECATS2 and the non-binding control. Despite this initial artifact, there is subsequently a clear and stark difference between the ECATS2 response and the non-binding control, supporting that ECATS2 reported a bona fide hypoosmotic stress-induced release of extracellular ATP.

**Figure 7:**
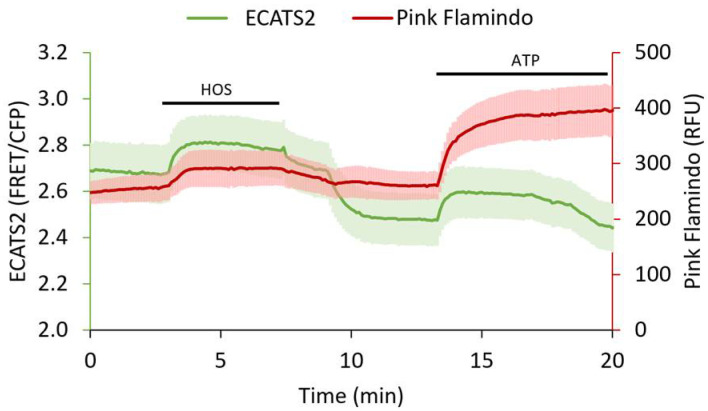
Three-color ECATS2 and Pink Flamindo dual biosensor imaging in P2Y_11_ receptor-expressing HEK293 cells. Hypoosmotic stress (HOS, black bar) caused ATP release reported by the ECATS2 sensitized FRET/CFP emission ratio (green, n = 3 cells, mean ± std). Concurrently, Pink Flamindo (red, n = 3 cells, mean ± std) increased intensity reported elevated cAMP levels. Both extracellular ATP and cAMP levels decreased upon return to isoosmotic solution, and responsivity of the sensors was validated with ATP wash in at the end of the experiment (ATP, black bar).

We also demonstrated that ECATS2 can be used simultaneously with another biosensor. In response to hypoosomotic stress, P2Y G-protein coupled receptors are thought to participate in volume regulation [5,22,38,39]. We therefore co-expressed ECATS2 and the red fluorescent cAMP biosensor Pink Flamindo [41] in HEK293 cells with co-expression of the P2Y_11_ receptor. The P2Y_11_ receptor is G_α,s_-coupled and has an EC_50_ of approximate 10 µM for ATP activation [10]. Upon hypoosmotic stress, the ECATS2 sensitized FRET emission ratio increased as expected. Furthermore, in P2Y_11_-expressing cells we also observed a concurrent transient increase in the Pink Flamindo intensity that reports the expected increase in cAMP levels (Figure 7). Thus, ECATS2 can be paired with other color-compatible biosensors to interrogate purinergic signaling.

**Figure 8:**
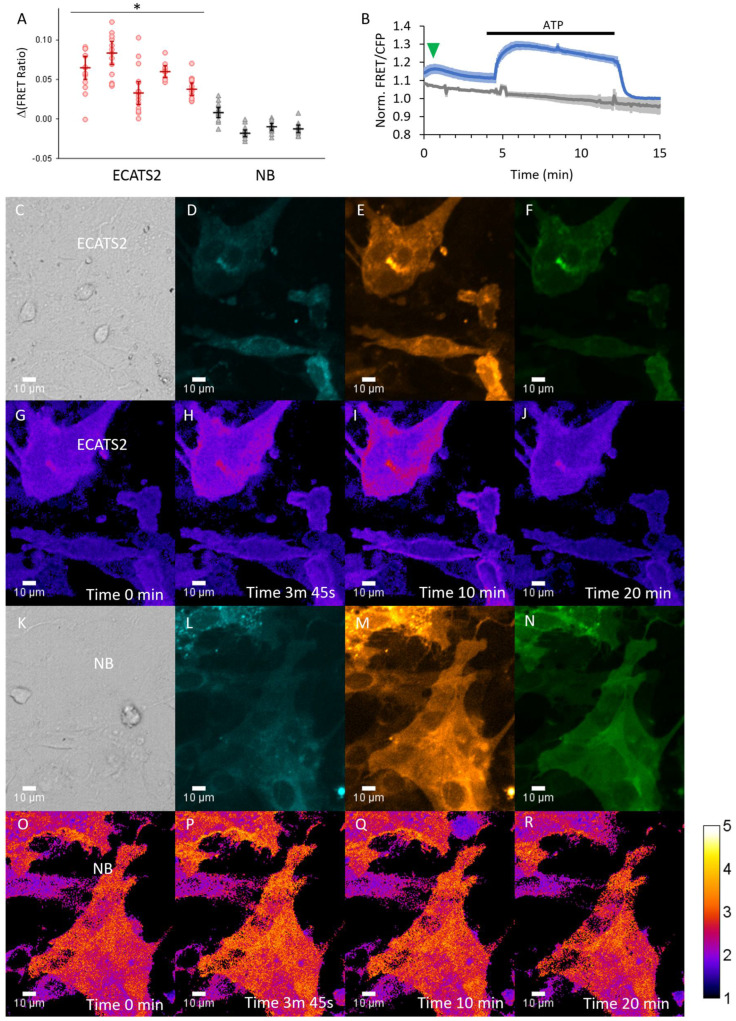
ECATS2 reports hypoosmotic stress-induced ATP release from cultured primary mouse cortical astrocytes. (**A**) Summary of experiments across independent primary cultures. Individual circles, cells per astrocyte culture imaged. Bars are median ± 95% confidence interval. * *p* < 0.001, 2-tailed Student’s *t*-test ECATS2 (red) versus non-binding NB control (black). Example trial: (**B**) Average response from cells expressing ECATS2 (blue, n = 13 cells) versus the non-binding NB control mutant (gray, n = 11 cells). After equilibration in isotonic solution, hypoosmotic solution was washed in at the start of the experiment, eliciting a clear response from ECATS2 (green arrowhead) absent from the NB control. To verify responsiveness and reversibility, ATP was washed in at 4 min and washed out at 12 min (black bar). Examples of (**C**–**J**) ECATS2 or (**K**–**R**) NB-expressing astrocytes with images for (**C**,**K**) DIC, (**D**,**L**) cyan, (**E**,**M**) yellow, (**F**,**N**) FRET channels as well as (**G**–**J**,**O**–**R**) ratio images pseudo-colored to the sensitized FRET emission ratio at the different time points indicated.

Lastly, we tested if ECATS2 can detect ATP release from primary astrocytes. Cultured mouse cortical astrocytes were transduced with adenovirus to express ECATS2 or the non-binding control mutant in separate cultures, and they were subjected to hypoosmotic stress. ECATS2 responded with a clear and reproducible increase in the sensitized FRET emission ratio (Figure 8). The ECATS2 response was starkly different from the lack of response of the non-binding mutant. We validated biosensor responsivity with the wash in of excess 200 µM ATP, demonstrating the reversibility of ECATS2 and the true non-response of the non-binding control mutant. Across several primary cultures, ECATS2 reproducibly responded to hypoosmotic stress while the non-binding control mutant did not respond, supporting that ECATS2 was reporting a bona fide release of extracellular ATP from astrocytes (Figure 8).

## 4. Discussion

We have demonstrated that ECATS2 has improved affinity for extracellular ATP and can be used to detected stimulated ATP release from live cells. In the engineering of ECATS2, we found that optimization of the surface tether was critical. In this study as well as our previous work developing ecATeam3.10, we observed that tethering a soluble biosensor to a cell surface attenuated its dynamic range and affinity, and the same phenomenon was observed with the iATPSnFR biosensors [28,29]. We hypothesized that dampened sensing could result from conformational restriction caused by the physical proximity to the cell membrane. We therefore used semi-rigid α-helical ER/K linkers to increase the length of the surface tether. This proved key to recovering some of the affinity gain imparted by the R103A/R115A double mutation, but it is also clear there is not a simple relationship between distance and performance. ECATS2 contains the 20 nm ER/K linker, whereas the 10 nm linker exhibited dampened sensing and the 30 nm ER/K linker did not further improve ATP affinity over the 20 nm linker. It is possible that the 10 nm linker is not long enough to increase conformational freedom. The 30 nm linker may be too long so that linker bending permits other interactions that also dampen sensing or that dilution of extracellular ATP is significantly greater at this distance from the membrane. In any case, these observations illustrate that optimization of the membrane tether is an important consideration for this type of cell-surface biosensor. In the future, it may be interesting to test whether optimizing a tether could facilitate the transformation of other ATP sensors such as iATPSnFR [29,31] or the MaLions [42] into extracellular ATP sensors, especially given that membrane-targeted iATPSnFR suffered from a similar dampening in sensing that ecATeam3.10 suffered [29]. It would also be interesting to explore whether an optimized tether could further improve other currently existing extracellular sensors, such as those for extracellular lactate, serotonin, nicotine, or acetylcholine to name a few [43,44,45,46].

We further observed that surface targeting of the original ecATeam3.10, the ecATeam.3.10(A/A) double mutant, DM-10, ECATS2, and DM-30 was similar for both Neuro2A and HEK293 cell lines, indicating that the longer tethers do not interfere with trafficking. Because these sensors use fluorescent proteins, they still suffer from background intracellular fluorescence caused by maturation of the fluorophores *en route* to the surface as described previously [28]. However, we were careful to isolate the surface signal during image analysis. Interestingly, targeting in primary mouse astrocytes was excellent. Regardless, the problem of intracellular background fluorescence may be a good reason to explore the use of chemigenetic-based sensors [44,47] in the future because of the availability of some cell-impermeant fluorophore labels.

We also demonstrated that ECATS2 can detect ATP release from live cells, not just the exogenous application of ATP. Our use of hypoosmotic stress was motivated by the role of purinergic signaling in cell volume regulation and pathologies such as injury-induced edema. We further showed that ECATS2 can be used simultaneously with a red fluorescent cAMP biosensor, illustrating that ECATS2 can be generally useful for correlating upstream primary events with downstream secondary signaling dynamics.

In comparison to other extracellular ATP biosensors, ECATS2 has some advantages and disadvantages. One advantage is that ECATS2 is the only intrinsically ratiometric extracellular ATP biosensor, while the iATPSnFR^1.0/1.1^ and GRAB_ATP1.0_ biosensors are single-color and require a reference fluorophore for ratiometric measurements. With its ability to detect as low as 0.5 µM extracellular ATP, ECATS2 has >35-fold higher affinity than iATPSnFR^1.1^. ECATS2 also exhibits a greater than 25-fold selectivity for ATP over ADP, whereas GRAB_ATP1.0_ does not distinguish between the nucleotides. However, ECATS2 has the major disadvantage suffered by many FRET biosensors that its dynamic range is orders of magnitude smaller than the single-color cpGFP biosensors, which is a major determinant of sensitivity and limit of detection. When considering ex vivo and in vivo experiments, the significantly larger response dynamic range has proven key to the success of the GRAB_ATP1.0_ sensor, which has been used in vivo in mice and fish for example [9,48,49]. Here, we found that tether length optimization is a key factor in tuning apparent ligand affinity, but we did not observe improvement in dynamic range. The soluble ATeam sensors exhibit much larger dynamic ranges than either ecATeam3.10 or ECATS2, suggesting that it should still be possible to improve the sensors in this aspect. In the future, the type of tether, structured or unstructured, may be worth exploring, but no matter the approach it is clear that further engineering will be necessary to increase the dynamic range and improve the practical applicability of this type of surface-tethered FRET sensor.

## Data Availability

Data is available upon request. Plasmid constructs are distributed by Addgene.

## Supplementary Materials

The following supporting information can be downloaded at: https://www.mdpi.com/article/10.3390/s25185903/s1, Figure S1: Structure showing residues R103 and R115; Figure S2: Cell mixing strategy for imaging; Figure S3: Mg2+-independence; Figure S4: DM-20 images; Figure S5: DM-30 example single-cell responses; Figure S6: ADP response.
