# Supplementary material for: An Improved Ratiometric FRET Biosensor with Higher Affinity for Extracellular ATP
Source: Sensors (Basel). 2025 Sep 21;25(18):5903. doi: 10.3390/s25185903 (PMC12473246; doi:10.3390/s25185903)
Supplement: Supplementary file 1 [file sensors-25-05903-s001.zip › sensors-3849322-supplementary.pdf]

An Improved Ratiometric FRET Biosensor with Higher Affinity for Extracellular ATP

Autumn Cholger [1,‡], Jason M. Conley [1,‡], Elaine Colomb [1], Olivia de Cuba [2], Jacob Kress [2], Mathew Tantama [2,*]

[1] Purdue University, Department of Chemistry & Interdisciplinary Life Science Program, West Lafayette, Indiana, USA
[2] Wellesley College, Department of Chemistry & Biochemistry Program, Wellesley, Massachusetts, USA
‡ These authors contributed equally to this work.
* Correspondence: mt4@wellesley.edu

Supporting Information

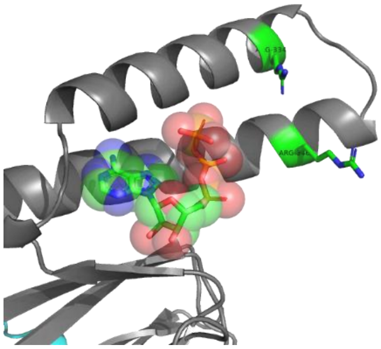

Figure S1. Zoomed in view of Figure 1 showing the R103 and R115 residues highlighted in green, which are mutated in the R103A/R115A double mutant.

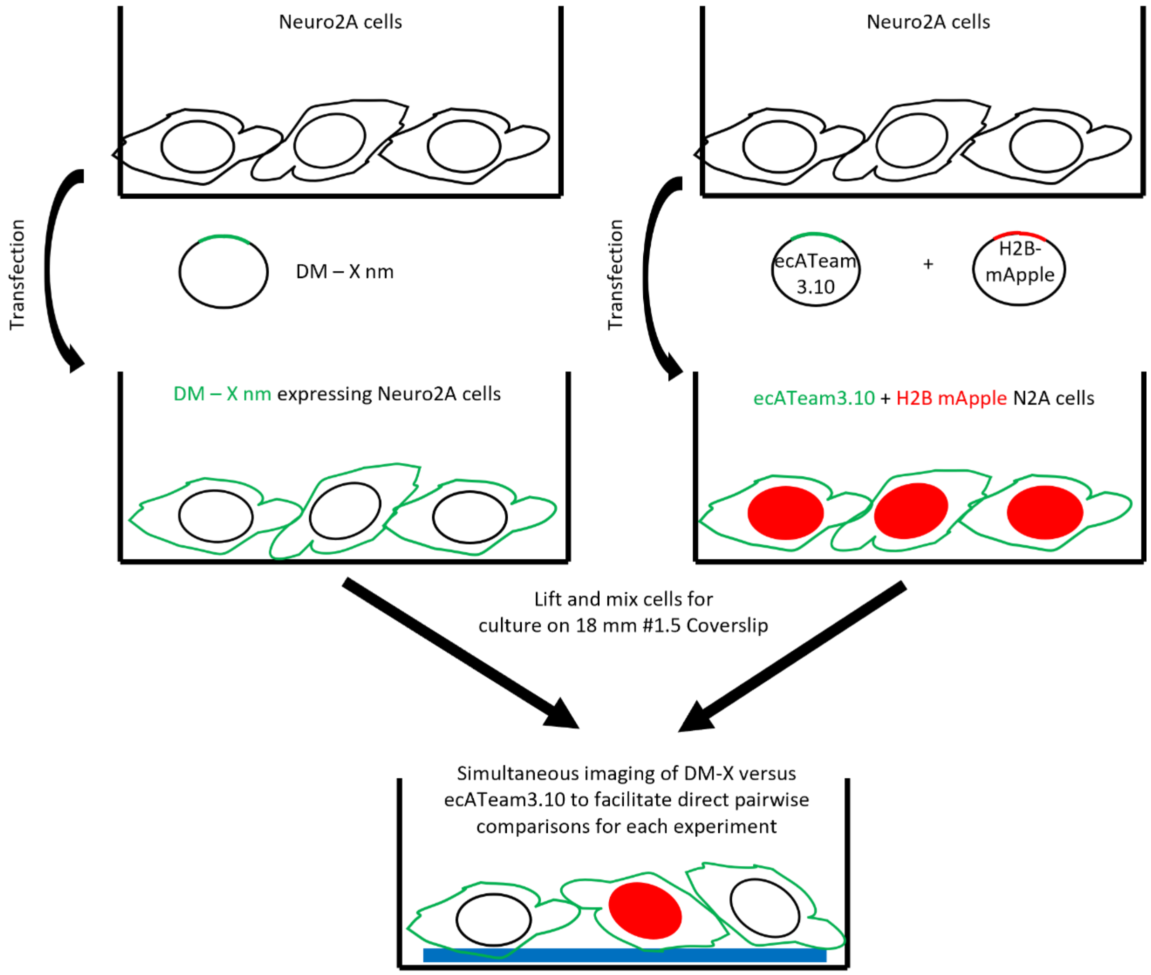

Figure S2. Cell mixing strategy for simultaneous imaging of a biosensor variant and the original ecATeam3.10 to facilitate direct pairwise comparison within each experiment. One set of cells was transfected with the ecATeam3.10(A/A) double mutant (DM) or double mutant plus tether length (DM-X nm) variant. A second set of cells was co-transfected with the original ecATeam3.10 and excess H2B-mApple to ensure all ecATeam3.10 expressing cells co-expressed H2B-mApple as a marker for identification. The two sets of cells were then lifted and mixed for culture on glass coverslips for simultaneous imaging under the same experiment conditions.

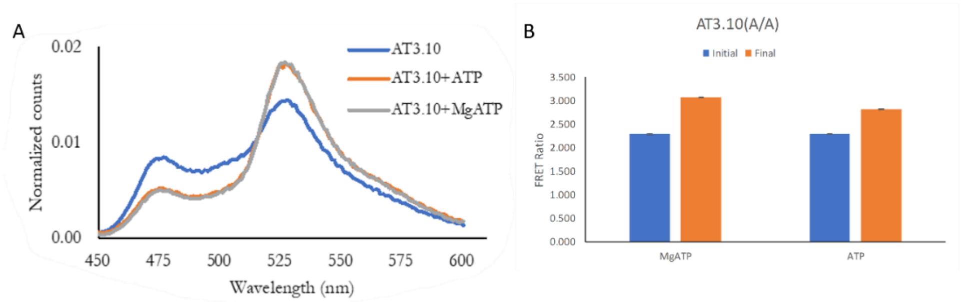

Figure S3. Mg^{2+}-Independent ATP binding for (A) ATeam3.10 and (B) ATeam3.10(R105A/R115A). In order to best control Mg^{2+} concentrations, the purified proteins of the soluble biosensor modules were assayed in solution.

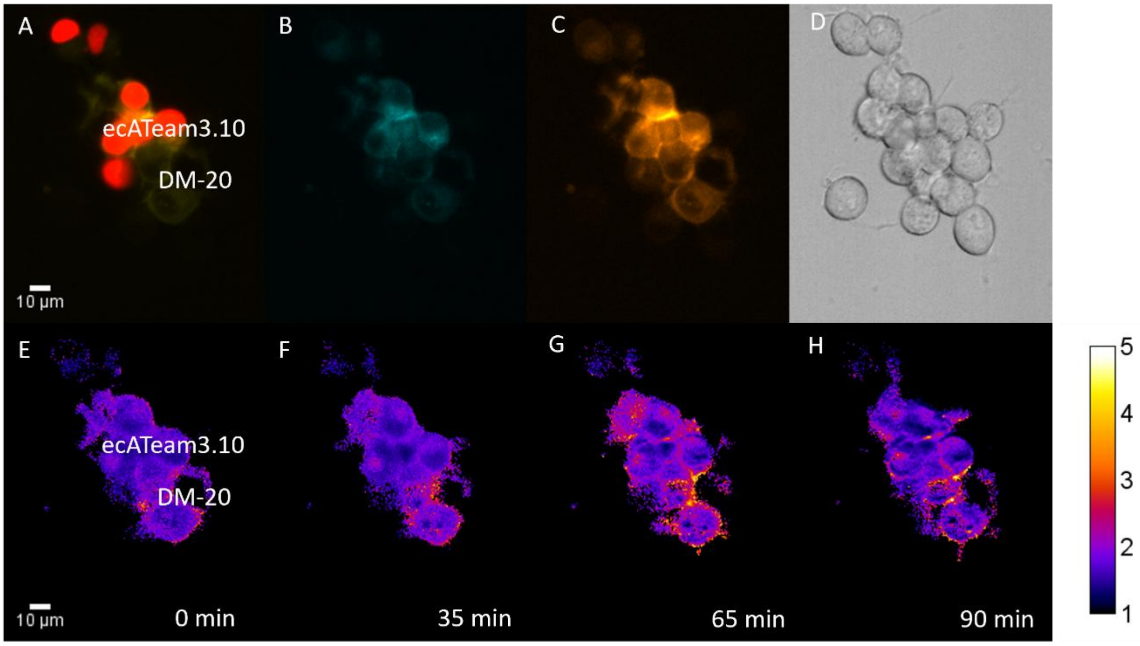

Figure S4. Example images for simultaneous imaging of DM-20 (ECATS2) expressing HEK293 cells versus cells expressing the original ecATeam3.10. Surface targeting efficiency was similar for the original ecATeam3.10, ecATeam3.10(A/A), DM-10, DM-20, and DM-30 in HEK293 and Neuro2A cells. Cells expressing the original ecATeam3.10 co-express H2B-mApple as a marker for identification. (A) Overlay of red, cyan, and FRET emission channels. (B) Cyan donor channel. (C) FRET emission channel. (D) DIC image. (E-H) ratio images at the indicated time points, pseudo-colored to show the sensitized FRET/CFP donor emission ratio.

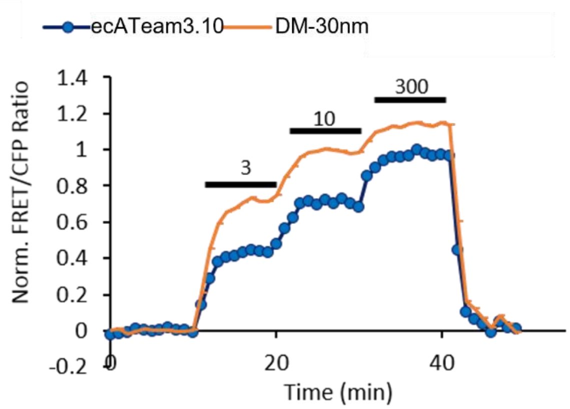

Figure S5. Example single-cell traces for the comparison of the original ecATeam3.10 sensor (blue) and the DM-30 tether length variant (orange). Extracellular ATP was perfused at the micromolar concentrations indicated by the black horizontal bars. Washout at the end of the experiment brought the sensitized emission ratio back to baseline, demonstrating reversibility. The DM-30 variant showed greater sensitivity compared to ecATeam3.10 at low ATP concentration. Although the DM-30 variant seemed to trend to a slightly higher dynamic ratio ($\Delta F/F_0$), the magnitude of the maximum response at saturating ATP for the average of all experiments was not statistically different from the dynamic range of the original ecATeam3.10.

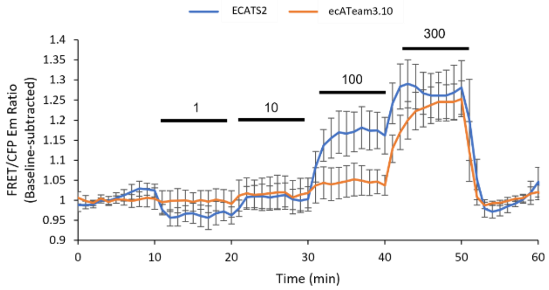

Figure S6. ADP Response. As described in Figure S2, ECATS2-expressing cells were mixed with ecATeam3.10 and H2B-mApple co-expressing cells to enable simultaneous imaging for direct comparison. The micromolar concentrations of ADP were washed in by perfusion as indicated by the black horizontal bars. As expected, ECATS2 showed increased affinity for ADP compared to the original ecATeam3.10. However, even with higher affinity, ECATS2 did not respond to 10 μM ADP and required upwards of 100 μM of ADP for a response, showing it still maintains greater sensitivity to ATP at physiologically relevant concentrations.
